# A Recovery Capital and Stress-Buffering Model for Post-deployed Military Parents

**DOI:** 10.3389/fpsyg.2018.01832

**Published:** 2018-10-04

**Authors:** David S. DeGarmo, Abigail H. Gewirtz

**Affiliations:** ^1^Department of Educational Methodology, Policy, and Leadership, Prevention Science Institute, University of Oregon, Eugene, OR, United States; ^2^Department of Family Social Science and Institute of Child Development, Institute for Translational Research in Children’s Mental Health, University of Minnesota, Minneapolis, MN, United States

**Keywords:** military families, parents, PTSD, recovery capital, social support, efficacy, intervention and prevention

## Abstract

We tested a recovery capital model for military families employing the After Deployment, Adaptive Parenting Tools (ADAPT) randomized control trial, a longitudinal preventive intervention study of 336 post-deployed military parents. Recovery resources included measures of social capital (parenting support, observed partner support behaviors), personal capital (parenting efficacy, education), and community capital (the ADAPT behavioral parent-training intervention). We hypothesized higher levels of recovery capital would buffer the negative impact of military stress on growth in post-traumatic stress disorder (PTSD) symptoms for deployed and civilian parents. Outcome data were evaluated with three waves across 2-years. Hypotheses were tested with latent growth models in a structural equation modeling framework. Military stress was assessed by reports of exposure to combat and battle aftermath. Recovery capital was measured by reported support for parenting and direct observation of behavioral interactions during problem-solving discussions of deployment-related stressors. Fathers had higher levels of military-related stress and PTSD symptoms over time compared to mothers. Growth curve models showed that fathers were characterized by individual differences in 2-year average levels of PTSD symptoms while mothers were characterized by individual differences in initial status and linear growth trajectories. Results supported a recovery capital model. Higher levels of parenting efficacy and parenting support were associated with lower PTSD symptoms, representing common pathways for both mothers and fathers. Similarly, parenting support operated as a moderating buffer for both parents. That is, effects of military trauma exposure on psychological distress were lower for mothers and fathers with higher levels of parenting support relative to parents with lower levels. Regions of significance indicated that half a standard deviation above the mean of support was beneficial for mothers, while one and half standard deviations were needed to impact the effects of trauma on fathers’ PTSD. For mothers assigned to the ADAPT parent training intervention – but not fathers – the intervention was associated with linear reductions in PTSD symptoms over 2 years. The recovery capital model explained 36% of the variance in father outcomes and 46% for mothers. The intervention obtained a medium effect size in reducing mothers’ symptoms (*d* = 0.41). Implications for prevention and treatment within a recovery capital model are discussed.

## Introduction

The recovery capital model was first developed in the substance use and addictions treatment field (Cloud and Granfield, 2004; White and Cloud, 2008); defined as the “depth and breadth of internal and external resources” one accesses to initiate and sustain recovery from psychological and behavioral maladies (Kelly and Hoeppner, 2015, p. 9). More simply put, recovery capital is the total sum of support resources one can garner during the recovery process (Cano et al., 2017). As individuals progress through their recovery journey toward the goal of complete resolution, recovery capital should increase, reducing the likelihood of remission (Kelly and Hoeppner, 2015).

Personal or “internal” recovery capital refers to intrinsic resources such as physical health and psychological traits that are associated with resilience including self-efficacy, problem solving abilities, knowledge, and education. “External recovery capital” includes resources that are external to the individual such as social relationships or community resources for promoting recovery that ameliorate personal maladies. Here, social relationships may be seen as more extant and naturalistic environmental resources. External resources also include more socially constructed resources (i.e., cultural and community resources) such as social policies, and available institutional services and treatment programs (White and Cloud, 2008).

In the present paper, we apply a recovery capital model to the study of psychological distress among post-deployed military parents. Evidence shows that deployment and combat stressors associated with military life predict family maladjustment (e.g., Sheppard et al., 2010; Card et al., 2011). For example, a growing body of research has shown links between deployment-related stress for civilian parents, and combat-related stress for deployed parents related to post-traumatic stress disorder (PTSD) and symptoms, and increased parenting difficulties (Gewirtz et al., 2010; Cozza, 2016). A primary consequence is the development and maintenance of PTSD symptomatology over time (Polusny et al., 2017).

To date, however, few studies have tested mechanisms explaining psychological adjustment of military parents using multiple method data. A majority of research on military populations has focused on soldier-only and soldier reported characteristics and outcomes. A more contextually valid view underscores military individuals as embedded within the context of their family relationships, family resources, and their communities (Gewirtz et al., 2011). Moreover, researchers in the recovery capital field have called for greater specificity of measurement and operational definitions of recovery resources as well as more empirical validation of these constructs (Cano et al., 2017). In this paper, we will test a longitudinal recovery capital model using both an experimental design and multiple method measurement of recovery capital resources.

## Post-Traumatic Stress and Recovery Capital for Military Parents

**FIGURE 1:**
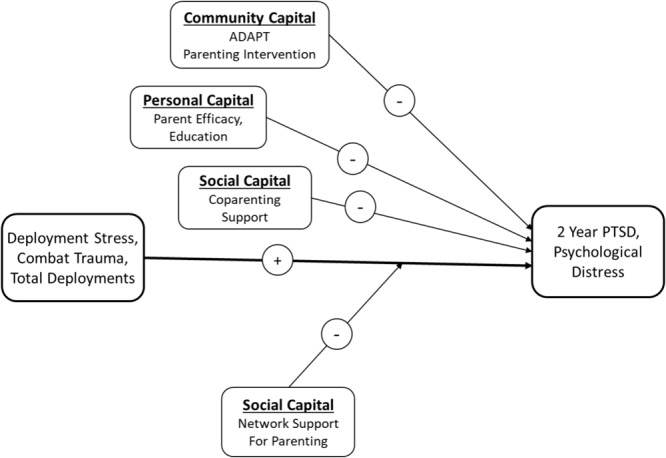
Hypothesized recovery capital and stress-buffering model for 2 year psychological distress of mothers and fathers following parental deployment.

We propose a recovery capital model using a set of well-defined internal and external resources for military parents. We test a stress-buffering model hypothesizing community, personal, and social capital as buffers to deployment-related stress and combat-related trauma of both civilian and deployed military parents. Our recovery capital model is shown in **Figure 1**. This figure is not proposed as a fully comprehensive set of possible pathways and resources, rather, it is a contextual model based on the breadth and scope of the data collected in the present study which were hypothesized protective factors for military families.

The top pathway of the model is conceptualized as *community capital*. We hypothesize that participation in a parent training intervention will be associated with reductions in PTSD symptoms over time. Merely belonging to a military family is not a mental health risk factor; however, the deployment and reintegration of a parent are a known family stressor associated with increased risk for adjustment difficulties for children, the active duty and post-deployed service member, and the at-home partner (Kelley and Jouriles, 2011). As Gewirtz and colleagues have noted (2011) for families with a deployed parent, there is anxiety over long separations, worries about a safe return, and interrupted communication. After reunification, there is elation at a parent’s return and new challenges to face during reintegration, especially when a veteran returns with psychological or physical injuries. Following reintegration, disruptions in parental functioning due to parental PTSD, psychological distress, and substance use predict children’s developmental adjustment problems and interfere with continued effective parenting (Gewirtz et al., 2017b).

The After Deployment, Adaptive Parenting Tools (ADAPT) study was the first randomized controlled trial to test a parent training program designed to reduce stress, improve emotion regulation, and to promote more effective behavioral and emotional parenting behaviors. Recent evidence has demonstrated that participation in the ADAPT program was associated with 1-year improvements in directly observed parenting practices measured by indicators of problem solving skill, discipline, monitoring, skill encouragement, and positive involvement, with a moderate effect size (*d* = 0.35) (Gewirtz et al., 2017a). In the prior 1-year evaluation, parenting practices were the primary and proximal target of the intervention. In this paper, we will focus on parent psychological distress as an outcome over 2 years. To provide a better test of recovery capital, we will also focus on baseline levels of recovery capital prior to randomization in the experimental design.

The second pathway is conceptualized as *personal capital*, operationalized here as parenting efficacy and educational attainment. A recent 1-year evaluation of the ADAPT intervention impact showed that participation in the parent training program was associated with improvements in 6-month parenting efficacy measured by the parental locus of control scale (Campis et al., 1986). Improvements in parenting efficacy, in turn, were associated with reductions in 1-year psychological distress and suicidal ideation for both mothers and fathers (Gewirtz et al., 2016). The ADAPT program obtained medium effect sizes on mothers’ efficacy and small effects on fathers’ efficacy. The second personal resource is educational attainment. Education and health literacy are known protective factors for mental and physical health outcomes. Prospective evidence shows that educational attainment is associated with reductions in psychological distress (Brännlund and Hammarström, 2013) and more specifically, reductions in PTSD following military trauma (Shalev et al., 1996).

The last recovery pathway in the model is through *social capital*, specifying main effects for spouse/partner support and a moderating buffer of parenting support networks. Gewirtz et al. (2017b) showed that supportive behaviors observed during co-parenting problem-solving discussions were concurrently associated with effective parenting practices in military families. Both experimental and non-experimental studies of parents at-risk for compromised parenting have utilized “hot topic” problem-solving paradigms in which parents attempt to resolve stressful co-parenting and personal issues. Higher levels of supportive behaviors during these discussions are predictive of greater parenting adjustment for both mothers (Forgatch and DeGarmo, 1997) and fathers (DeGarmo and Forgatch, 2012). We hypothesize a main effect additive buffer for observed co-parenting support on psychological distress. We also tested hypotheses for mothers and fathers separately because of known gender differences in behavioral parenting practices, differences in support behaviors and seeking, and traditional gender role expectations often supported by military culture (Dunkel-Schetter and Skokan, 1990; DeGarmo, 2016).

Finally, we propose that perceived social support for parenting will moderate the negative impact of military stress on psychological distress for post-deployed parents, also known as the “stress-buffering” framework (Schwarzer and Leppin, 1991; Thoits, 1995). The buffering model is predicated on studies of health and stress indicating a person’s appraisal of support tends to moderate or mitigate the effects of stressors. In the face of stressful events and processes, those garnering high levels of support are buffered from the detrimental impact of stress; conversely, those who are more insular and garner lower levels of support are more likely to experience distress outcomes. The large majority of social support research has tested models of perceived support. Little research has focused on support for parenting needs specifically (cf., DeGarmo et al., 2008).

## Hypotheses

In summary, based on the recovery capital model conceptualized as the sum of internal and external resources that promote an ongoing pathway to recovery, we will test a longitudinal adjustment model for psychological distress of deployed and civilian parents. We include a family systems perspective on parent adjustment using multiple method assessment of recovery capital in domains of personal capital, social capital, and community capital (the latter, in the form of a preventive intervention program). We recognize the model in **Figure 1** is not an exhaustive set of recovery resources that are relevant for military parents. Nonetheless, to our knowledge this is the first multiple method randomized trial evaluating a contextual recovery capital model for parents. To expand prior 1-year evaluations of the ADAPT program, we will focus on baseline recovery capital resources as buffers to adjustment over time. Because the sample included a mother or father who was deployed, we use the terms military fathers and military mothers hereafter to include deployed or civilian parents from military-related families and refer to deployed parents specifically where appropriate.

Based on the literature reviewed above, we formulated the following hypotheses:

Community capital hypothesis: Parents receiving the ADAPT parent training intervention will exhibit lower levels of PTSD over 2 years relative to the control condition.
Personal capital hypothesis: Parents with higher levels of parenting efficacy and education will exhibit lower levels of PTSD over 2 years.
Social capital hypothesis: In the face of deployment and combat-related stressors, parents with high levels of social support for parenting will be buffered from the negative effects of stress; conversely, parents with low levels of support will be more vulnerable to the negative impact of stress on PTSD symptoms over 2 years.

## Materials and Methods

The current sample included parents from 336 military families; 314 mothers and 294 fathers. Participants consented and completed a baseline assessment for a prevention study evaluating the effectiveness of a parenting program (ADAPT). Families were eligible to participate in the study if at least one parent had deployed to recent conflicts (i.e., Operation Iraqi Freedom or Operation Enduring Freedom, OIF/OEF) and at least one child between the ages of 4 and 12 years was living in the home.

All participants gave written informed consent. Informed consent procedures and all assessment protocols were in accordance with the ethical standards of the 1964 Helsinki Declaration and its later amendments, and with the ethical standards of the American Psychological Association. Consent procedures and assessment protocols were approved by the University of Minnesota and University of Oregon institutional review boards for the protection of human participants.

Of the 336 families participating in the study, 272 families had two parents participating in the study and 64 families had one parent participating. Among the two-parent families, 258 couples were married to each other, 12 couples were not married to each other, and 2 did not indicate marital status. Of the 64 parents participating in the study without a partner, 41 were mothers and 23 were fathers. Parents participating alone reported being married (but husband declined study participation; *n* = 23), divorced (18), single (10), separated (9), or widowed (1). The rest did not indicate marital status (*n* = 3). Number of children in a household ranged from 1 to 6 with a mean of 2.34 children in a family (*SD* = 0.96). Following the baseline assessment, 60% of the parents were randomized to the intervention (ADAPT condition) and 40% to a services-as-usual control condition. The latter provided family “tip sheets” and online parenting resources. The ADAPT program is a 14-week multi-family group program targeting improvement of emotion regulation and parenting skills via active teaching methods such as role play, practice, and discussion (Gewirtz et al., 2014). Groups of 4–15 parents and two to three facilitators met weekly for 2 h in a convenient community location (college, church, community center, etc.); dinner was served and childcare provided. The program was web-enhanced, such that all parenting skills, handouts, and mindfulness practices were available online in short video vignettes.

Participants were predominantly White (88.4% of fathers and 92.7% of mothers). Mothers’ ages ranged from 23 to 51 years (*M* = 35.67, *SD* = 5.89), and fathers’ ages ranged from 23 to 58 years (*M* = 37.75, *SD* = 6.54). About half of participants (47.7% of fathers and 51.9% of mothers) reported completing at least a Bachelor’s degree. Household incomes ranged from $39,999 or less (13.8%) to $120,000 or more (14.5%), with most families reporting income between $40,000–$79,999 (43.5%) and $80,000–$119,999 (28.2%). Most fathers (84.3%) and about half of mothers (48.4%) were employed full-time.

In 86.7% of participating families one parent was deployed to recent conflicts; and in the remaining 13.3% of families, both parents were deployed to recent conflicts. In 18.2% of families, the mother was deployed; in 95% of the families, the father was deployed. Most parents deployed with the Army National Guard (59%); others deployed with the Air National Guard (10.7%), the Army (12.9%), Navy (6.6%), Air Force (2.8%), or Marine Reserves (0.3%). During these operations, 51.2% of deployed parents were deployed more than once for an average of 1.73 deployments (*SD* = 1.16). In 58.3% of these deployments parents were deployed for more than 12 months. According to Hoge et al.’s (2014) criteria, 16% of fathers and 7% of mothers reported clinical levels of PTSD symptom severity.

Of the 314 mothers and 294 fathers who completed the baseline assessment, 255 (81%) mothers and 226 (76.8%) fathers completed the 1 Year assessment (T3), and 249 (80%) mothers and 219 (74%) fathers completed the 2 Year Assessment (T4)^1^. For family participation, 82% of the study sample was retained for in-home or online assessments (85 and 80%, respectively, for control and intervention), there was no differential attrition by study condition for enrolled families. There were no significant differences in most demographics (i.e., race, income, age, marital status, number of children) between mothers and fathers who completed the T4 assessment and those who dropped out. However, mothers who completed the T4 assessment (*M* = 2.27, *SD* = 0.59) had more children on average than mothers who dropped out (*M* = 2.54, *SD* = 1.21) as measured at baseline [*t*(307) = -2.05, *p* < 0.05]. After the baseline assessment (T1), 60% of the families were randomized to the ADAPT parent training condition and 40% to the services as usual control condition. Psychological distress outcome data were evaluated at three time points across a 2-year study period, baseline, 1- and 2-year follow-ups (T1, T3, and T4).

## Measures

### Stress and Distress Measures

#### Combat-Related Stress and Trauma

Two subscales of the Deployment Risk and Resilience Inventory (DRRI-2: King et al., 2006) were employed to measure military trauma: the 15-item *Combat Exposure* scale and the 15-item exposure to *Battle Aftermath* scale. Parents retrospectively reported their deployment experiences at T1 in response to the stem, “*Thinking about your combat experiences during deployment, please circle ‘yes’ if the statement is true and ‘no’ if the statement is false*.” Each scale was a summative index of items endorsed yes. Sample items for the combat scale were: *I went on combat patrols, … encountered water mines, booby traps, … received hostile incoming fire, … unit suffered casualties, etc.* The T1 Kuder–Richardson (KR-20) alpha reliability for dichotomous items was 0.87 and 0.83, for fathers and mothers, respectively. Sample items for battle aftermath were: *I was exposed to the sight, sound, or smell of dying men and women, … was involved in removing dead bodies after battle, … took care of injured or dying people, … saw the bodies of dead enemy soldiers, … saw the bodies of dead civilians, etc.* The KR-20 alpha was 0.91 and 0.90, for fathers and mothers, respectively.

#### Number of Deployments

The number of deployments indexed the total count of overseas deployments.

#### Post-traumatic Stress Disorder (PTSD)

Psychological distress was measured with the PTSD Symptom Checklist (PCL; Hoge et al., 2014) military and civilian versions, a 17-item questionnaire of post-traumatic stress as defined by the DSM-IV. Deployed parents responded to the stem, *Below is a list of problems and complaints that veterans sometimes have in response to a stressful military experience.* Civilian parents responded to the stem, *Below is a list of problems and complaints that people sometimes have in response to stressful life experiences.* All items were rated on a five-point Likert scale from 1 (*not at all*) to 5 (*extremely*). Sample items were; *feeling irritable or having angry outbursts, … trouble falling or staying asleep, … disturbing memories, thoughts or images of a stressful military experience, … feeling distant or cut off from other people, … feeling very upset when something reminded you of a stressful military experience, … loss of interest in things that you used to enjoy, etc*. (Cronbach’s α ranged from 0.95 to 0.96 from T1 to T3 for fathers, and from 0.91 to 0.93, for mothers).

### Recovery Capital Measures

#### Intent to Treat (ITT)

The preventive intervention effect was coded 1 for random assignment to the ADAPT intervention condition and 0 for control condition.

#### Parent Education

Educational attainment was measured in categories ranging from 1 (*some high school or less*) to 8 (*doctoral or professional degree; Ph.D., M.D., D.D., J.D., etc.*).

#### Parenting Efficacy

Efficacy was measured with the Parental Locus of Control Short Form Revised/PLOC – SFR (Hassall et al., 2005) a shortened form of the Parental Locus of Control Scale (Campis et al., 1986). The 24-item SRF self-report questionnaire measures parenting control orientation (i.e., internal vs. external) in four dimensions: parental efficacy, parental responsibility, child control of parents’ life, and parental control of child’s behavior. Participants rated the items on a five-point Likert scale from 1 (*strongly agree*) to 5 (*strongly disagree*). Sample items were: *What I do has little effect on my child’s behavior*; *My child’s behavior is sometimes more than I can handle*; *When something goes wrong between me and my child, there is little I can do to correct it*; *When I make a mistake with my child I am usually able to correct it*; *Parents should address problems with their children because ignoring them won’t make them go away.* A total mean score was obtained, with high scores indicating high sense of control, efficacy, or internality and low scores indicating low efficacy (T1 father α = 0.73 and mother α = 0.75).

#### Problem Solving

Parents’ problem solving skill was measured using the problem solving outcome measure of parenting practices at baseline (T1), i.e., the parents’ ability to resolve and negotiate solutions to parent–child issues. Scores were obtained from direct observation of parent–child interactions during structured Family Interaction Tasks (FITs). FITs included three problem-solving tasks requiring parent(s) and child to address current conflicts, a monitoring task, and two teaching tasks, lasting 40 min. In prior studies, FIT codes demonstrated ecological validity, construct validity, and sensitivity to change with at-risk families (Forgatch and DeGarmo, 1999; Gewirtz et al., 2009). Trained coders scored FITs using the Coder Impressions system (Forgatch et al., 1992) slightly modified for the current study (Zamir et al., 2017). The Coder Impressions system is a global rating, macro-level, coding system designed to assess parenting practices, including verbal and nonverbal behaviors. Coders individually watch each task and make an overall rating based on intensity, frequency, and duration of each summary code (e.g., empathy, respect, positive reinforcement, etc.). *Problem solving* skill was scored with a nine-item scale rated on a five-point Likert scale from 1 (*untrue*) to 5 (*very true*), evaluating the quality of parent’s and child’s solution, extent of resolution, satisfaction at the outcome of the discussion, and likelihood the family would put this solution to use (α = 0.87 for fathers and 0.88 for mothers at T1).

#### Co-parenting Behavioral Support

Support from co-parent was assessed with a 5-min conflict discussion adapted from Bullard et al. (2010) and administered as part of the FITs. Partners were asked to rate in order of severity current conflicts related to co-parenting and deployment (e.g., getting back on the same page after deployment). Couples were instructed to attempt to solve the conflict rated as most severe. Couples were left alone during the interactions, which were videotaped. Couples’ communication was assessed with the Positive Communication Scale (Zamir et al., 2017), a nine-item scale assessing expression of humor, affection, empathy, interest in the partner, agreement, positive affect, positive involvement, and engaged body posture. Sample items are: “*Showed empathy and genuine concern*,” “*Used humor in a friendly and supportive way, to set at ease or lighten the situation*,” “*Was verbally affectionate with partner*.” Coders rated duration, intensity, and frequency of behaviors on a six-point Likert scale ranging from 1 (*never*) to 6 (*always*). Items were averaged to create summary scores, such that higher scores indicated higher positive communication. Alphas were 0.77 for fathers and 0.76 for mothers. Observers underwent biweekly reliability meetings to minimize observer drift. Inter-rater reliability was assessed for 15% of the videos with different randomly selected coders teams. *ICCs* were 0.92 for fathers’ and 0.91 for mothers’ positive communication.

#### Parenting Support

Social support for parenting needs was measured at T1 with the Parenting Support Index (PSI: DeGarmo et al., 2008) a 24-item index rated on a five-point Likert scale from 0 (*not at all/not applicable*) to 4 (*a great deal*). Parents reported the amount of support received within four domains: emergency child care (e.g., *if you get sick, have appointments, or have to work overtime*), nonemergency child care (e.g., *need time to do something fun or relaxing*), practical parenting assistance (e.g., *advice, doctor referrals, help with doctor appointments, driving to and from daycare/school*), and financial assistance with parenting. Each domain was answered for six different relationship types: Current spouse/partner, relative(s), friend(s), neighbor(s), coworker(s), and former spouse/partner. The total index score was the sum of items in each domain (Cronbach’s T1 α was 0.89 and 0.90 for fathers and mothers, respectively).

## Analytic Strategy

**FIGURE 2:**
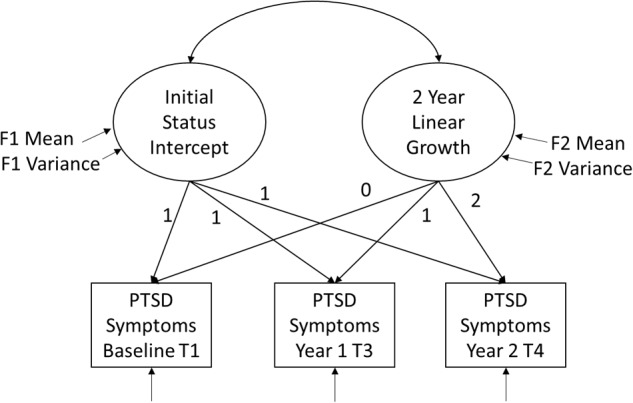
Specification of unconditional latent growth model for PTSD symptoms over 2 years.

Analyses were conducted in two stages. The first stage was to characterize the pattern of growth in PTSD symptoms for fathers and mothers using linear growth curve modeling. The second stage tested the recovery capital model. Hypotheses were tested with structural equation modeling (SEM) in M*plus* 8 (Muthén and Muthén, 1998–2017). Longitudinal PTSD data were specified as latent growth models (LGM), a special case of multi-level modeling in the SEM framework in which repeated measure outcomes at Level 1 are nested within individuals at Level 2. More specifically, the first stage of growth analysis estimates an unconditional growth model as shown in **Figure 2**. Using criteria recommended by Duncan et al. (2006), two latent factors are estimated with the repeated measures observed variables defining the latent factor means and variances using fixed chronometric time weights of 1, 1, and 1 for the initial status intercept factor; and 0, 1, 2 for the linear growth or the trajectory slope factor. Criteria focus on whether the latent factor means or sample summaries are significantly different from zero, and whether there are significant factor variances, meaning there are individual differences in levels or trajectories. Should both factors obtain significant variances (i.e., individual differences in levels or change slopes) and there is adequate fit to the data, then both factors are retained. If the linear growth factor does not obtain significant variance and no mean sample growth, then the growth factor is dropped, reducing the model to an “average level” or random intercept over time model. Maximum-likelihood SEM model fit was evaluated with recommended criteria (Kline, 2010) including a non-significant chi-square minimization *p*-value, a comparative fit index above 0.90, and a root mean square error of approximation (RMSEA) lower than 0.06.

The next stage of analyses, the conditional model, enters hypothesized predictors of PTSD over time. To test the cumulative recovery capital model we evaluated independent effects or additive effects of the recovery resources. Because the majority of fathers were deployed parents and the majority of mothers were civilian parents, all models were tested separately for mothers and fathers. Moreover, models were estimated using full information maximum likelihood (FIML; Brown et al., 2008), which uses all available information from the observed data in the SEM analyses. FIML estimates are computed by maximizing the likelihood of a missing value on the basis of observed values in the data. Compared with mean-imputation, list-wise, or pair-wise models, FIML provides more statistically reliable standard errors (Brown et al., 2008). Individuals who have baseline data only and no follow-up data contribute nothing to the likelihood of estimates for growth and are effectively excluded from longitudinal analyses; however, they contribute to the covariance of data collected at Time 1. Further, because models control for the number of deployments and military stressors, only those individuals deployed contribute to the estimation of combat-related stressors; whereas individuals with recovery capital (civilian and military) contribute to the estimation of hypothesized buffering pathways. Likewise, only parents who have co-parents contribute to the estimate of co-parenting support behaviors. Although these approaches can be problematic when missingness is non-ignorable, they are still less biased and recommended for SEM (Graham, 2003; Brown et al., 2008).

Finally, to test the support buffering moderating effect, the model was specified as a special case of maximum likelihood that can estimate latent variable interactions. First, social support for parenting was entered as a standardized and centered first-order term and the centered cross product of parenting support × military trauma was specified as a latent variable interaction in MPlus using maximum likelihood with robust standard errors (MLR) and a numerical integration algorithm (Muthén and Muthén, 1998–2017). This random effects model estimates the effect of latent trauma on PTSD at varying levels of parenting support.

## Results

The means, standard deviations, *n* sizes, and mean comparisons for the key study variables are shown in **Table 1**. Fathers and mothers did not differ in levels of education or parenting efficacy. Fathers and mothers also exhibited similar levels of observed support behaviors toward partners during the problem solving discussions. For military-related stressors and measures of distress, fathers in the sample were significantly higher in PTSD symptoms at each of the three time points, were higher in combat exposure, and marginally higher in the battle aftermath score. Fathers also reported higher levels of parenting support from social network relationships relative to mothers in the sample.

**Table 1:** Means, standard deviations, *N*s, and mean comparisons for study variables.

	Fathers			Mothers			
Variable	*N*	*M*	*SD*	*N*	*M*	*SD*	*t*
Education	287	5.17	1.29	309	5.28	1.26	0.97
Parenting efficacy	285	3.66	0.40	305	3.62	0.42	1.23
Partner support behaviors	245	3.10	0.69	245	3.16	0.71	0.91
Deployments overseas	293	3.25	1.75	306	1.29	0.71	17.80^∗∗∗^
PTSD symptoms T1	287	29.97	12.39	305	27.24	9.59	2.98^∗∗∗^
PTSD symptoms T3	226	28.60	11.99	252	25.88	9.27	2.75^∗∗^
PTSD symptoms T4	212	28.93	12.25	249	26.86	9.53	1.96^∗^
Combat exposure T1	274	4.59	3.66	53	3.15	2.91	2.71^∗∗^
Battle aftermath T1	274	4.89	4.48	53	3.62	4.14	1.82^†^
Parenting support T1	287	68.76	10.53	245	66.47	11.41	2.53^∗^

*^∗∗∗^*p* < 0.001, ^∗∗^*p* < 0.01, ^∗^*p* < 0.05, ^†^*p* < 0.10.*

Turning to the first stage of the primary analyses, we estimated the unconditional growth model for parent PTSD over time. Results of the best fitting growth factors are presented in **Table 2** by parent. The father PTSD model did not provide adequate fit to retain a linear growth factor. That is, there was no significant mean change for the sample, nor was there significant variance or individual differences in growth trajectories over time. Thus, the best fitting model reduced to a random intercept or “2-year average levels” model of PTSD [χ^2^(4) = 5.63, *p* = 0.23, CFI = 0.99, RMSEA = 0.03]. The random intercept model indicated that there were significant individual differences in the average levels of PTSD over 2 years for the fathers. This means the father PTSD trajectories were rather stable, or chronically high or low over time. On average fathers’ PTSD total score on the total PCL was 29.47 over 2 years (*p* < 0.001).

**Table 2:** Latent variable means, variances, and standard errors for best-fitting 2-year unconditional growth model of PTSD symptoms by parent.

	Fathers			
Latent growth factor	Mean	*SE*	Variance	*SE*
2-year average levels PTSD (Intercept)	29.47^∗∗∗^	0.68	120.14^∗∗∗^	11.37

**Latent growth factor**	**Mothers**			

Initial status PTSD (Intercept)	27.10^∗∗∗^	0.55	76.37^∗∗∗^	9.40
2-year linear growth PTSD (Slope)	-0.53^∗^	0.28	8.18^∗^	3.68

*Father model fit [χ^*2*^(4) = 5.63, *p* = 0.23, comparative fit index (CFI) = 0.99, root mean square error of approximation (RMSEA) = 0.03]. Mother mode fit [χ^*2*^(1) = 11.14, *p* = 0.001, comparative fit index (CFI) = 0.97, root mean square error of approximation (RMSEA) = 0.03]. ^∗∗∗^*p* < 0.001, ^∗∗^*p* < 0.01, ^∗^*p* < 0.05, ^†^*p* < 0.10.*

The best fitting model for mothers obtained significant initial status differences as well as differences in growth trajectories over time as indicated by the variance components shown in **Table 2** [χ^2^(1) = 11.14, *p* = 0.001, CFI = 0.97, RMSEA = 0.03]. On average the sample of mothers scored 27.10 on the PCL at baseline (*p* < 0.001), and in addition, the mothers in the sample showed a mean drop of -0.53 per year (*p* < 0.01). The variance component for the growth factor indicated that some mothers were increasing and some mothers were decreasing over time.

**FIGURE 3:**
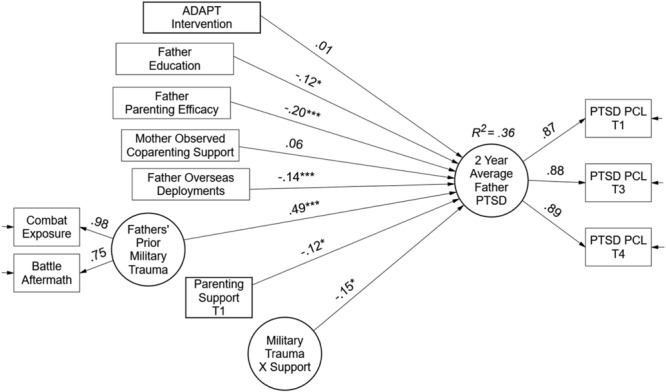
Test of recovery capital model for military fathers. Paths are standardized coefficients [free parameters = 19; –2LL = –3261.05; Akaike Information Criteria (AIC) = 6560.09; and Bayesian Information Criteria (BIC) = 6625.90].

The second stage of the analyses tested the hypothesized recovery capital model for fathers and mothers. There was strong support for a recovery capital model across parents. Hypotheses were supported for fathers on the personal and social capital variables, and hypotheses were supported for mothers with regards to the community, personal, and social capital domains. Results of the MLR latent variable interaction model are presented in **Figure 3** for fathers in the form of standardized path coefficients.

The right-hand side of the father model specifies the random intercept, or average levels of PTSD over 2 years. The military trauma factor was a strong predictor of PTSD levels (β = 0.49, *p* < 0.001). Contrary to expectations, more deployments were associated with lower levels of PTSD symptoms (β = -0.14, *p* < 0.01). Support for the community capital hypothesis was not found in terms of the ADAPT intervention at the top of the model. However, support was obtained in the personal and social capital domains of the model. Both father education and parenting efficacy were associated with lower levels of PTSD over 2 years (Education β = -0.12, *p* < 0.05, and Efficacy β = -0.20, *p* < 0.01). For social capital, partner support behaviors were not associated with PTSD; however, support for parenting obtained both a significant main effect and moderating effect. The main effect in the presence of the interaction term represents the effect of parenting support at average levels of trauma (β = -0.12, *p* < 0.05); meaning, on average, parenting support was protective for PTSD symptoms among fathers. The moderating effect provided evidence of a stress-buffering model. That is, the latent variable interaction (Trauma × Support β = -0.15, *p* < 0.05) indicated the negative effect of military trauma was moderated or buffered by parenting support such that higher levels of support were associated with lower impact of trauma on average PTSD symptoms; and conversely, when support was low, trauma exposure had a stronger effect on PTSD symptoms. Overall, the recovery capital model explained 36% of the variance in fathers’ PTSD symptoms.

**FIGURE 4:**
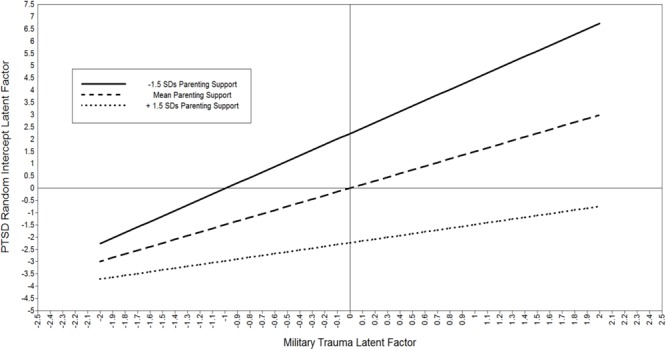
Plot of moderating effect of parenting support on the negative impact of trauma on PTSD symptoms for military fathers. Both the trauma factor and PTSD factor are centered latent variables; zero represents the factor mean. The estimated “region of significance” and bootstrapped confidence intervals indicated that parenting support reduced trauma’s impact starting at 1.4 standard deviations of support for fathers. That is, above 1.4 standard deviations there is a non-significant effect of Trauma on PTSD.

To illustrate the buffering effect of support, estimands were plotted in Mplus using loop plots (Muthén et al., 2016). The moderating effect of parenting support is shown in **Figure 4**. Both the trauma factor on the *X*-axis and the PTSD symptoms average level factor on the *Y*-axis are centered latent variables; therefore, zero represents the factor mean. Simple slopes for the effect of trauma are estimated from the regression coefficients for -1.5 SDs, the mean, and +1.5 SDs in parenting support. The estimated “region of significance” and bootstrapped confidence intervals indicated that parenting support reduced trauma’s impact starting at 1.4 standard deviations of support for fathers. That is, above 1.4 standard deviations above the mean the effect of trauma exposure on PTSD symptoms was non-significant.

**FIGURE 5:**
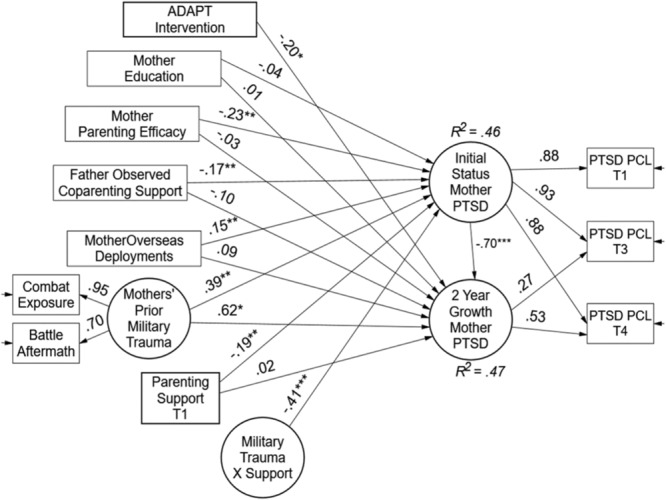
Test of recovery capital model for military mothers. Paths are standardized coefficients [free parameters = 28; –2 LL = –2871.27; Akaike Information Criteria (AIC) = 5798.55; and Bayesian Information Criteria (BIC) = 5901.88].

Results of the MLR latent variable interaction model for mothers are shown in **Figure 5** in the form of standardized path coefficients. On the right-hand side of the model, both initial status PTSD symptoms and 2-year linear growth are represented by the latent variable factors. Starting at the top of the model, the ADAPT intervention program was associated with reductions in maternal PTSD symptoms over time (β = -0.20, *p* < 0.05). Mothers’ parenting efficacy was associated with lower levels of T1 PTSD symptoms (β = -0.23, *p* < 0.01) but education was not. Fathers’ behavioral support during problem solving discussions predicted lower levels of T1 PSTD (β = -0.23, *p* < 0.01). As expected, the greater the number of prior deployments, the higher the T1 PTSD symptoms, while prior trauma exposure reported by those mothers who deployed predicted both initial status and linear increases in PTSD over 2 years (β = 0.39, *p* < 0.01 and β = 0.62, *p* < 0.05, respectively). Like the fathers’ model, parenting support obtained an average level main effect and a moderating effect on initial levels of PTSD symptoms for mothers. At average levels of trauma exposure, support had a beneficial effect (β = -0.19, *p* < 0.01), and further, support moderated the negative impact of trauma (β = -0.41, *p* < 0.001). Overall, the recovery capital model explained 46% of mothers’ initial PTSD symptoms and 47% of the variance in linear growth.

**FIGURE 6:**
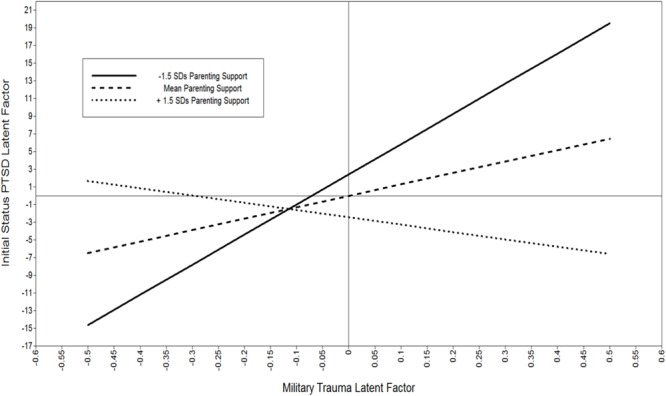
Plot of moderating effect of parenting support on the negative impact of trauma on PTSD symptoms for military mothers. Both the trauma factor and PTSD factor are centered latent variables; zero represents the factor mean. The estimated “region of significance” and bootstrapped confidence intervals indicated that parenting support reduced trauma’s impact starting at 0.45 standard deviations of support for mothers. That is, above half a standard deviations there was a non-significant effect of Trauma on PTSD.

**FIGURE 7:**
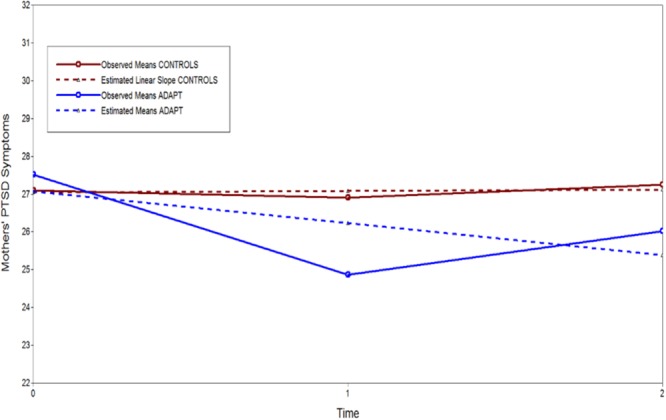
Observed means and unconditional linear growth plot of ADAPT intervention effect on 2-year growth in PTSD for military mothers.

Two plots are illustrated to show the effects of recovery capital for mothers in the sample. The first plot (**Figure 6**) shows the moderating effect of parenting support. The estimated region of significance and bootstrapped confidence intervals indicated that parenting support reduced trauma’s impact starting at 0.45 standard deviations of support for mothers. That is, above half a standard deviation beyond the mean there was a non-significant effect of trauma exposure on PTSD symptoms. Finally, the intervention impact on reductions in PTSD over time is plotted in **Figure 7** shown as the observed means and the estimated unconditional linear growth for both mothers assigned to the control condition and mothers assigned to the ADAPT intervention. The final model indicated that controlling for other recovery capital domains, the ADAPT intervention was associated with a medium effect reducing maternal psychological distress (effect size = 0.41).

## Discussion

We tested a recovery capital model for military families following parental deployment. We employed multiple domains of internal and external recovery resources hypothesized to promote improvements in mental health over time. We conceptualized personal capital as parenting efficacy and education; social capital was defined as observed support behaviors from co-parenting partners; and unique to a randomized controlled trial, we conceptualized the ADAPT parenting program as a domain of community capital. Because we measured both behavioral support and perceived parenting support, our model incorporated recovery capital from a stress-buffering framework with observed support transactions hypothesized to obtain main effects and perceived support hypothesized to obtain moderating effects of stress. To our knowledge, this is the first evaluation of recovery resources for military families within the context of a randomized controlled trial.

**FIGURE 8:**
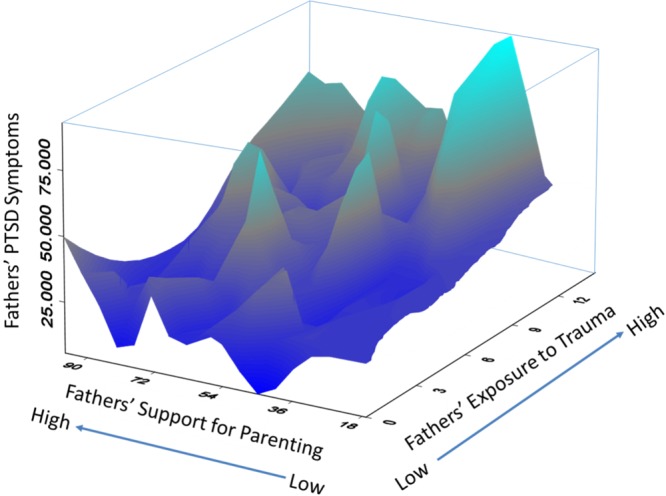
3D surface plot of observed data for fathers in military families. Y-axis is the 2-year average PTSD symptoms, the X-axis is fathers’ exposure to military trauma, and the Z-axis is fathers’ social support for parenting needs. The buffering effect is of support is represented in the upper left corner and risk is represented in the upper right corner (hight trauma and low support).

Either parent or both parents in the present sample had previously deployed. Comparing coupled and uncoupled fathers with mothers showed that fathers had higher levels of military-related stress and PTSD symptoms over time compared to the mothers in the study, which is unsurprising given that almost all fathers (compared to just 57 mothers) had deployed. Despite these mean level differences there were some common pathways of recovery for both fathers and mothers, as well as unique pathways. Common pathways for both mothers and fathers were obtained for measures of parenting efficacy and parenting support as additive buffers to PTSD. That is, for all parents, perceptions of greater parenting efficacy and support were associated with lower average levels or growth in PTSD symptoms. Similarly, parenting support operated as a moderating buffer for both mothers and fathers. That is, the effects of military trauma exposure on psychological distress were lower for mothers and fathers with higher levels of parenting support relative to parents with lower levels. Consistent with the, on average, higher and more stable levels of PTSD symptoms for fathers, the region of significance indicated that only half a standard deviation above the mean of support was beneficial for mothers, while one and half standard deviations of support was needed to impact fathers’ PTSD symptoms. These findings are consistent with a large body of literature indicating the importance of social support as a buffer for both the development and maintenance of PTSD (e.g., Guay et al., 2006; Adams et al., 2017). Further research is needed to understand the nuances of this issue – for example, the implications of differential support as capital for diverse levels of PTSD symptoms, as well as for different sociodemographic factors (age, gender, SES, etc.). A conceptual and empirical model emphasizing the importance of social capital is presented in **Figure 8** using a 3D surface plot for the father data. The *Y*-axis is the 2-year average PTSD symptoms, the *X*-axis is fathers’ exposure to military trauma, and the *Z*-axis is fathers’ social support for parenting needs. The buffering effect is of support is represented in the upper left corner and risk is represented in the upper right corner (high trauma and low support).

Unique pathways for fathers versus mothers were obtained for education, behavioral support, and the ADAPT parenting program. Higher levels of education benefited fathers but not mothers, while behavioral support from partners benefitted mothers and not fathers. The education findings are consistent with prior findings that elements of socio-economic status appear to be important protective factors for fathers’ postdeployment family functioning (Davis et al., 2015) and that mothers are more affected by fathers’ support – and their psychological health – than vice-versa (Zamir et al., 2017). Finally, the other unique pathway for mothers was the parent training program. For mothers assigned to the ADAPT parenting program – but not fathers – the intervention was associated with direct reductions in PTSD symptoms. This community capital effect for mothers was illustrated in **Figure 7**.

These findings are consistent with the notion that fathers in the present sample had higher levels of PTSD symptoms and likely need more intensive dosage and more specified cognitive behavioral therapy to directly address PTSD. However, we also note, as discussed in the introduction, prior findings from the effectiveness trial showed that improvements in mother and father parenting efficacy as a result of the program were related in turn to reductions in PTSD symptoms in both parents (Gewirtz et al., 2016). Further analyses are needed to determine whether there may be other potential indirect effects of the ADAPT program on fathers’ PTSD symptoms.

The primary limitation of the current study was the lack of data across the deployment cycle. All data were gathered following parental deployment(s). Future studies should examine recovery capital using prospective longitudinal data gathered prior to as well as following parental deployment (cf., Polusny et al., 2017). Another limitation was the predominantly White study population (88 and 93%, respectively, for mothers and fathers). Recent estimates show that 17% of current US veterans are African American and 12% Hispanic (Bialik, 2017). More research is needed to determine if recovery models are more salient for racial and ethnic veterans. A key strength of the current study was the use of a systems perspective to define and measure a recovery capital model for military families that including multiple time points, methods, and informants. The cumulative recovery capital model was also evaluated as independent effects or additive buffers, that is, the cumulative regression estimates in the final path models. Future investigations could better examine subpopulation or empirically derived classes of participants using cumulative resource indices of recovery capital. Our findings provide a foundation from which to further examine personal, social, and community recovery capital in the context of exposure to traumatic stress.

### Implications

Applying and evaluating a recovery capital model proved useful for illustrating common and unique pathways to psychological recovery for military-related mothers and fathers. Common pathways showed that exposure to combat-related stressors was harmful to both mothers and fathers and that parenting support provided direct benefits as well as buffering benefits on psychological distress. These data suggest that recovery capital within the naturalistic social environment (immediate and extended) is a vital resource. Other effective sources of psychological recovery are programmatic resources and community services. Prior evaluation of the ADAPT parent training program evinced improved parenting behaviors for mothers and fathers. The present evaluation showed the program’s effective impact on psychological recovery for mothers; however, more intensive services are likely needed for fathers. Given higher levels of PTSD symptoms, fathers may be more vulnerable to in-theatre combat exposure, battle aftermath, and potential injury. Better tests of the unique pathways will require larger samples of military exposed mothers.

Developmental research shows that fathers, in general, also behave less sensitively to their children, engage in more rough and tumble play, and form less close attachments with their children compared to mothers (DeGarmo, 2016). Given the non-normative challenges of military families that place fathers and mothers at risk for ineffective parenting during and following the deployment cycle, services must address the requirements of families in general and unique factors for the deployed parent. For naturalistic parenting supports, it is critically important in supporting deployed parents to prepare and maintain connection to their families. Examples of preparatory family activities include “*… creating family journals, scrapbooks, or electronic journals and webpages before deployment; establishing Skype accounts and reassuring children by planning distance routines; checking and planning for operational security issues that may prevent parents from receiving or sending pictures, videos, and audio; discussing fears and emotions with children of appropriate age*” (DeGarmo, 2016, p. 57). Other family activities include activities focused on resources for financial planning and legal documents (e.g., power of attorney, wills, emergency care, and contact plans).

For clinical implications and professional resources, clinicians need to consider the full deployment cycle rather than reintegration solely. Stress during pre-deployment can include anticipatory separation anxiety. During the course of the deployment cycle, the civilian parents (more likely mothers) are dealing with lengthy separation and sole parenting. The deployed parents (typically fathers) try to balance absence from the family and staying connected to their children. Issues during family reintegration can also emerge in reestablishing a parenting “equilibrium” upon the service member’s return (Gewirtz et al., 2011, p. 60). Clinicians should emphasize resilience and strength-based approaches for treatment. For example, pointing out ways in which the family has successfully negotiated challenges in the past by confronting, growing, and learning from other significant military or more general stressors is an appropriate strategy for building upon extant parenting skills and effective parenting practices and family communication. Focusing on what is working and setting goals, rather than focusing on deficits or problems, sets up families for future successes, and effective maintenance of recovery.

## Author Contributions

DD and AG contributed theoretical conception and design of the study, and wrote the manuscript. DD performed the statistical analysis.

## Conflict of Interest Statement

The authors declare that the research was conducted in the absence of any commercial or financial relationships that could be construed as a potential conflict of interest.

## Footnotes

1 There was an immediate post-test assessment, T2, at 6 months post-baseline, but data from T2 are not included in this paper because that assessment point did not include a measure of observed parenting practices, or some measures of parents’ psychological distress.

## References

[B1] Adams R. E. Urosevich T. G. Hoffman S. N. Kirchner H. L. Hyacinthe J. C. Figley C. R. (2017). Social support, help-seeking, and mental health outcomes among veterans in non-VA facilities: results from the Veterans’. Health Study. *Mil. Behav. Health* 5 393–405. 10.1080/21635781.2017.1333067 29098116 PMC5663244

[B2] Bialik K. (2017). *The Changing Face of America’s Veteran Population.* Washington, DC: Pew Research Center Available at: http://pewrsr.ch/2jgY89s

[B3] Brännlund A. Hammarström A. (2013). Higher education and psychological distress: a 27-year prospective cohort study in Sweden. *Scand. J. Public Health* 42 155–162. 10.1177/1403494813511559 24265167

[B4] Brown H. C. Wang W. Kellam S. G. Muthén B. O. Petras H. Toyinbo P. (2008). Methods for testing theory and evaluating impact in randomized field trials: intent-to-treat analyses for integrating the perspectives of person, place, and time. *Drug Alcohol Depend.* 95(Suppl. 1) S74–S104. 10.1016/j.drugalcdep.2007.11.013 18215473 PMC2560173

[B5] Bullard L. Wachlarowicz M. DeLeeuw J. Snyder J. Low S. Forgatch M. S. (2010). Effects of the Oregon model of Parent Management Training (PMTO) on marital adjustment in new stepfamilies: a randomized trial. *J. Fam. Psychol.* 24 485–496. 10.1037/a0020267 20731495 PMC2928579

[B6] Campis L. K. Lyman R. D. Prentice-Dunn S. (1986). The parental locus of control scale: development and validation. *J. Clin. Child Psychol.* 15 260–267. 10.1207/s15374424jccp1503_10

[B7] Cano I. Best D. Edwards M. Lehman J. (2017). Recovery capital pathways: modelling the components of recovery wellbeing. *Drug Alcohol Depend.* 181 11–19. 10.1016/j.drugalcdep.2017.09.002 29028554

[B8] Card N. A. Bosch L. Casper D. M. Wiggs C. B. Hawkins S. A. Schlomer G. L. (2011). A meta-analytic review of internalizing, externalizing, and academic adjustment among children of deployed military service members. *J. Fam. Psychol.* 25 508–520. 10.1037/a0024395 21707171

[B9] Cloud W. Granfield R. (2004). A life course perspective on exiting addiction: the relevance of recovery capital in treatment. *NAD Publication Nordic Council Alcohol Drug Res.* 44 185–202.

[B10] Cozza S. J. (2016). “Parenting in military families faced with combat-related injury, illness, or death,” in *Parenting and Children’s Resilience in Military Families* eds Gewirtz A. H. Youssef A. M. (New York, NY: Springer Press) 151–173. 10.1037/ser0000038

[B11] Davis L. Hanson S. Zamir O. Gewirtz A. H. DeGarmo D. (2015). Associations of contextual risk and protective factors with fathers’ parenting practices in the post-deployment environment. *Psychol. Serv.* 12 250–260. 10.1037/ser0000038 26213794 PMC4591747

[B12] DeGarmo D. S. (2016). “Placing fatherhood back in the study and treatment of military fathers,” in *Parenting and Children’s Resilience in Military Families* eds Gewirtz A. H. Youssef A. M. (New York, NY: Springer International Publishing) 47–63. 10.1007/s10464-011-9437-y

[B13] DeGarmo D. S. Forgatch M. S. (2012). A confidant support and problem solving model of divorced fathers’ parenting. *Am. J. Commun. Psychol.* 49 258–269. 10.1007/s10464-011-9437-y 21541814 PMC3181268

[B14] DeGarmo D. S. Patras J. Eap S. (2008). Social support for divorced fathers’ parenting: testing a stress buffering model. *Fam. Relat.* 57 35–48. 10.1111/j.1741-3729.2007.00481.x 19177181 PMC2631442

[B15] Duncan T. E. Duncan S. C. Strycker L. A. (2006). *An Introduction to Latent Variable Growth Curve Modeling: Concepts, Issues, and Application* 2nd Edn. Mahwah, NJ: Lawrence Earlbaum Associates 10.1177/0265407590074002

[B16] Dunkel-Schetter C. Skokan L. A. (1990). Determinants of social support provision in personal relationships. *J. Soc. Pers. Relationsh.* 7 437–450. 10.1111/j.1467-9507.1997.tb00104.x 17854339

[B17] Forgatch M. S. DeGarmo D. S. (1997). Confidant contributions to parenting and child outcomes. *Soc. Dev.* 6 237–253. 10.1037/0022-006X.67.5.711

[B18] Forgatch M. S. DeGarmo D. S. (1999). Parenting through change: an effective prevention program for single mothers. *J. Consult. Clin. Psychol.* 67 711–724. 10.1037/a0016732 10535238

[B19] Forgatch M. S. Knutson N. M. Mayne T. (1992). *Coder Impressions of ODS Lab Tasks.* Eugene, OR: Oregon Social Learning Center.

[B20] Gewirtz A. H. DeGarmo D. S. Plowman E. J. August G. Realmuto G. (2009). Parenting, parental mental health, and child functioning in families residing in supportive housing. *Am. J. Orthopsychiatry* 79 336–347. 10.1037/a0016732 19839671

[B21] Gewirtz A. H. DeGarmo D. S. Zamir O. (2016). Effects of a military parenting program on parental distress and suicidal ideation: after deployment adaptive parenting tools. *Suicide Life Threat. Behav.* 46 S23–S31. 10.1111/sltb.12255 27094107 PMC5113712

[B22] Gewirtz A. H. DeGarmo D. S. Zamir O. (2017a). After deployment, adaptive parenting tools: 1-Year outcomes of an evidence-based parenting program for military families following deployment. *Prevent. Sci.* 19 589–599. 10.1007/s11121-017-0839-4 28913717 PMC5854502

[B23] Gewirtz A. H. DeGarmo D. S. Zamir O. (2017b). Testing a military family stress model. *Fam. Process* 57 415–431. 10.1111/famp.12282 28299783 PMC6788861

[B24] Gewirtz A. H. Erbes C. R. Polusny M. A. Forgatch M. S. DeGarmo D. S. (2011). Helping military families through the deployment process: strategies to support parenting. *Profess. Psychol.* 42 56–62. 10.1037/a0034134 21841889 PMC3155511

[B25] Gewirtz A. H. Pinna K. L. Hanson S. K. Brockberg D. (2014). Promoting parenting to support reintegrating military families: after deployment, adaptive parenting tools. *Psychol. Serv.* 11 31–40. 10.1037/a0034134 24564441 PMC4030517

[B26] Gewirtz A. H. Polusny M. A. DeGarmo D. S. Khaylis A. Erbes C. R. (2010). Posttraumatic stress symptoms among National Guard soldiers deployed to Iraq: associations with parenting behaviors and couple adjustment. *J. Consult. Clin. Psychol.* 78 599–610. 10.1037/a0020571 20873896 PMC3073229

[B27] Graham J. W. (2003). Adding missing-data-relevant variables to FIML-based structural equations models. *Struct. Equat. Model.* 10 80–100. 10.1207/S15328007SEM1001_4

[B28] Guay S. Billette V. Marchand A. (2006). Exploring the links between posttraumatic stress disorder and social support: processes and potential research avenues. *J. Trauma Stress* 19 327–338. 10.1111/j.1365-2788.2005.00673.x 16788995

[B29] Hassall R. Rose J. McDonald J. (2005). Parenting stress in mothers of children with an intellectual disability: the effects of parental cognitions in relation to child characteristics and family support. *J. Intellect. Disabil. Res.* 49 405–418. 10.1111/j.1365-2788.2005.00673.x 15882391

[B30] Hoge C. W. Riviere L. A. Wilk J. E. Herrell R. K. Weathers F. W. (2014). The prevalence of post-traumatic stress disorder (PTSD) in US combat soldiers: a head-to-head comparison of DSM-5 versus DSM-IV-TR symptom criteria with the PTSD checklist. *Lancet Psychiatry* 1 269–277. 10.1016/s2215-0366(14)70235-4 26360860

[B31] Kelly J. F. Hoeppner B. (2015). A biaxial formulation of the recovery construct. *Addict. Res. Theory* 23 5–9. 10.3109/16066359.2014.930132

[B32] Kelley M. L. Jouriles E. N. (2011). An introduction to the special section on U.S. military operations: effects on military members’ partners and children. *J. Fam. Psychol.* 25 459–460. 10.1037/a0024569 21842993

[B33] King L. King D. W. Vogt D. S. Knight J. Samper R. E. (2006). Deployment Risk and Resilience Inventory: a collection of measures for studying deployment-related experiences of military personnel and veterans. *Mil. Psychol.* 18 89–120. 10.1207/s15327876mp1802_1

[B34] Kline R. B. (2010). *Principles and Practice of Structural Equation Modeling* 3rd Edn. New York, NY: Guilford Press.

[B35] Muthén B. Muthén L. Asparouhov T. (2016). *Regression and Mediation Analysis using Mplus.* Los Angeles, CA: Muthén & Muthén.

[B36] Muthén L. K. Muthén B. O. (1998–2017). *Mplus User’s Guide* 8th Edn. Los Angeles, CA: Muthén & Muthén. 10.1002/jts.22199

[B37] Polusny M. A. Erbes C. R. Kramer M. D. Thuras P. DeGarmo D. Koffel E. (2017). Resilience and posttraumatic stress disorder symptoms in national guard soldiers deployed to Iraq: a prospective study of latent class trajectories and their predictors. *J. Trauma Stress* 30 351–361. 10.1002/jts.22199 28763565

[B38] Schwarzer R. Leppin A. (1991). Social support and health: a theoretical and empirical overview. *J. Soc. Pers. Relationsh.* 8 99–127. 10.1176/ajp.153.2.219 8561202

[B39] Shalev A. Y. Peri T. Canetti L. Schreiber S. (1996). Predictors of PTSD in injured trauma survivors: a prospective study. *Am. J. Psychiatry* 153 219–225. 10.1037/a0020332 8561202

[B40] Sheppard S. C. Malatras J. W. Israel A. C. (2010). The impact of deployment on U.S. military families. *Am. Psychol.* 65 599–609. 10.1037/a0020332 20822199

[B41] Thoits P. A. (1995). Stress, coping, and social support processes: where are we? What next? *J. Health Soc. Behav.* 53–79. 10.2307/2626957 7560850

[B42] White W. Cloud W. (2008). Recovery capital: a primer for addictions professionals. *Counselor* 9 22–27. 10.1177/0192513X17698182

[B43] Zamir O. Gewirtz A. H. Labella M. DeGarmo D. S. Snyder J. (2017). Experiential avoidance, dyadic interaction and relationship quality in the lives of veterans and their partners. *J. Fam. Issues* 39 1191–1212. 10.1177/0192513X17698182 PMC12380367 40874119

